# Peliosis hepatis mimicking cancer: A case report

**DOI:** 10.3892/ol.2013.1479

**Published:** 2013-07-19

**Authors:** WEIDONG DAI, DEWU ZHONG

**Affiliations:** Department of General Surgery, Xiangya Second Hospital, Central South University, Changsha, Hunan 410011, P.R. China

**Keywords:** peliosis hepatis, primary liver cancer, diagnosis

## Abstract

Peliosis hepatis (PH) is a rare condition characterized by the presence of blood-filled cavities within the liver. The etiology of PH remains unknown, but it has been reported to be associated with infections or malignancies. However, the cause of PH is not clear in 20–50% of patients. The current study presents the case of a 19-year-old male who presented with right upper quadrant pain that had lasted for three days. The patient was a student with no previous medical history. Contrast-enhanced computer tomography (CT) and ultrasonography showed a neoplasm in the right liver and a diagnosis of primary liver cancer was made due to the manifestation of the disease and the results of physical tests. The individual was treated successfully with an irregular right hemihepatectomy and was in good health at 6-months post-surgery. A tissue specimen was obtained and determined to be PH by pathological examination and immunohistochemistry analysis. Consequently, a diagnosis of PH must be considered in cases like this.

## Introduction

Peliosis hepatis (PH) is a rare condition characterized by blood-filled cystic cavities, ranging between 1 mm and several centimeters in diameter (1,2). The mechanism of PH is associated with sinusoidal expansion, which is caused by obstructions in the junction of the sinusoidal and central veins of the liver. This results in focal hepatic necrosis, liver sinusoidal barrier destruction and damaged endothelial cells, as red blood cells enter the space of Disse from the sinusoids and form cystic cavities (3). The current study presents the case of a 19-year-old male who complained of right upper quadrant pain that had lasted for three days. The patient was a student with no previous medical history. Contrast enhanced computer tomography (CT) and ultrasonography identified a neoplasm in the right liver, which was hypothesized to indicate primary liver cancer by the manifestation of the disease and the physical tests. The patient was treated successfully with an irregular right hemihepatectomy and was in good health at 6-months post-surgery. A tissue specimen was obtained and was determined to be PH by pathological examination and immunohistochemistry analysis. Written informed consent was obtained from the patient.

## Case report

**Figure 1:**
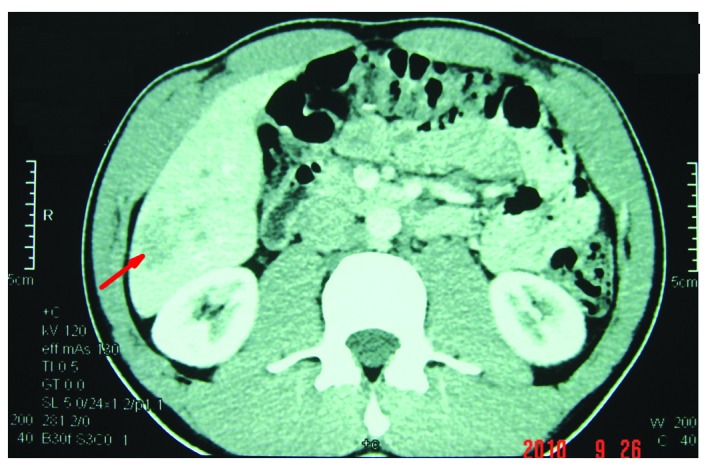
Computed tomography (CT) scan showing the Neoplasm (~3.5×4.5×4.5 cm) in the right lobe of the liver, as indicated by the arrow.

A 19-year-old male was admitted to Xiangya Second Hospital (Changsha, China) complaining of right upper quadrant pain that had lasted for three days. Enhanced CT and ultrasonography showed a neoplasm of ~3.5×4.5×4.5 cm in size in the right lobe of the liver (Fig. 1). The periphery of the neoplasm was significantly enhanced in the arterial phase. Laboratory tests were performed with the following results: White blood cell count, 4.8×10^9^/l (normal, 4–10×10^9^/l); hemoglobin, 155 g/l (normal, 110–160 g/l); hematocrit, 47.7% (normal, 39–52%); platelet count, 232×10^9^/l (normal, 100–300×10^9^/l); total bilirubin, 12.6 μmol/l (normal, 5.1–17.1 μmol/l); prothrombin time, 12 sec (normal, 11–15 sec); and α-fetoprotein, 2.01 mg/l (normal, <20 mg/l). In addition, electrocardiography and chest X-rays showed no marked abnormalities. The patient had no previous medical history and no history of exposure to toxic agents or drug use. Right upper quadrant pain lasting three days was the only manifestation and the degree of pain was slight, but persistent, and was not accompanied by fever or vomiting. The case was discussed with radiologists due to its specificity and a diagnosis of a hepatocarcinoma was suggested.

Following the pre-operative preparations, surgery was performed under general anesthesia. A mass was identified in the right lobe of the liver, but it did not resemble a hepatocarcinoma and the texture was soft. Mobilization of the right liver lobe was carried out by cutting the ligaments, and prior to cutting the mass, the Pringle maneuver was performed to prevent bleeding. As predicted, the intraoperative frozen pathology of the mass indicated that it was benign. The patient recovered well and was discharged one week later.

**Figure 2:**
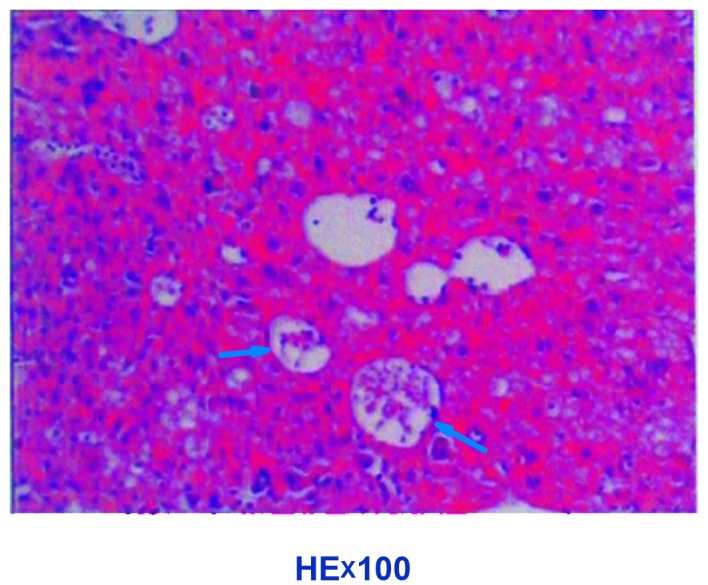
Microscopically-determined variably sized blood-filled cystic spaces and hemorrhagic necroses were present adjacent to peliotic spaces without endothelium lining, as indicated by the arrow.

The specimen was confirmed to be PH by pathological examination and immunohistochemistry analysis. Microscopically, there were blood filled cystic spaces of variable sizes and hemorrhagic necroses were present adjacent to peliotic spaces without endothelial lining (Fig. 2). Immunohistochemistry tests for CD31, CD34 and SMA were negative in the sinusoidal dilation area, but positive in the normal sinusoidal area.

## Discussion

PH was first described in 1861 by Wagner (4) and named by Schoenlank in 1916 (5). The etiology of PH remains unknown, but it has been reported to be associated with infectious and non-infectious causes, including drugs, chemicals, bacterial and viral infections and malignancies. *Bartonella henselae* is hypothesized to be the primary cause of infection (6) and Kitchell *et al*(7) previously demonstrated that PH is associated with *Bartonella henselae* in dogs. In addition, human immunodeficiency virus infection (8) and other wasting diseases (6,9–11) are associated with PH due to the patients weakened immune system, which directly or indirectly increases the risk of *Bartonella henselae* infection. However, a previous study reported that PH in cats is not associated with *Bartonella henselae* infection (12), indicating that cats have limited value as models for the analysis of *Bartonella henselae* in PH and that this association must be investigated further. The action of vascular endothelial growth factor has been observed to be important in the pathogenesis of PH (13). Drugs that act against PH include androgenic-anabolic steroids (3), tamoxifen (14), contraceptive steroids (15) and corticosteroids (16). Notably, PH may present as the cardinal symptom of specific diseases, including Hodgkin’s lymphoma (17). However, the causes of PH have not be identified in 20–50% of patients (18), as observed in the current case report.

Hepatocellular carcinoma (HCC) is one of the most common types of tumor worldwide, and particularly in China. Enhanced CT is the primary tool used to distinguish PH from HCC. Commonly, HCC shows hyperattenuation during the arterial phase, with rapid washout during the portal venous phase and iso- or hypoattenuation during the delayed phase. By contrast, during the arterial phase of PH, the lesions usually show early globular enhancement. In addition, multiple small accumulations of contrast material in the center and centrifugal progression of enhancement, without a mass effect on hepatic vessels, is present during the portal venous phase, as determined by enhanced CT. Diffuse increased attenuation may be observed during the delayed phase (18–20). Small lesions (diameter, >1 cm) may not be visible on enhanced CT (21) and magnetic resonance images are atypical for such lesions and the lesions may therefore be confused with hematomas, hemangiomas and HCC (2). PH may not be completely distinguished from HCC and other liver tumors by imaging tests. In the current case report, the patient had no previous medical history and enhanced CT showed a neoplasm mimicking cancer with atypical symptoms and a negative AFP value, making the formation of a diagnosis difficult. In addition, a biopsy was not performed as it could have caused a fatal hemorrhage.

An increasing number of studies have analyzed PH and possible differential diagnoses include hemangioma, hepatic adenoma, focal nodular hyperplasia, hepatic abscess and hypervascular metastases. In patients with atypical liver lesions, a diagnosis of PH must be considered, particularly in patients with no previous medical history or identifiable causes. Currently, there are no specific treatments available for PH, however, surgery must be performed on patients with a hemorrhage, long-term medical history or limited lesions. In addition, a liver transplant is necessary when patients have serious accompanying symptoms, including hepatic function failure. In these cases, the termination of any prescribed drugs is vital.

## Abbreviations

PH: peliosis hepatis
CT: computer tomography
